# *Helicobacter pylori* Infection: Comparison of Knowledge between Health Science and Non-Health Science University Students

**DOI:** 10.3390/ijerph18158173

**Published:** 2021-08-02

**Authors:** Taghreed A. Hafiz, Juliana Linnette D’Sa, Sahar Zamzam, Maria Liza Visbal Dionaldo, Murad A. Mubaraki, Regie Buenafe Tumala

**Affiliations:** 1 Clinical Laboratory Sciences Department, College of Applied Medical Sciences, King Saud University, Riyadh 12372, Saudi Arabia; mmubaraki@KSU.EDU.SA; 2 Maternal and Child Health Nursing Department, College of Nursing, King Saud University, Riyadh 12372, Saudi Arabia; jdsa@KSU.EDU.SA (J.L.D.); szamzam@KSU.EDU.SA (S.Z.); 3 Obstetric and Gynaecologic Nursing Department, Alexandria University, Alexandria 21544, Egypt; 4 Metro Technology Centers, Oklahoma City, OK 73111, USA; marializa.dionaldo@gmail.com; 5 Medical-Surgical Nursing Department, College of Nursing, King Saud University, Riyadh 12372, Saudi Arabia; rtumala@KSU.EDU.SA

**Keywords:** awareness, infectious disease, *H. pylori* infection, knowledge, undergraduate student, infection control, gastric cancer

## Abstract

*Background: Helicobacter pylori* (*H. pylori*), an important human pathogen, is classified as a human carcinogen. It is known to cause dyspepsia, peptic ulcers, and gastric cancer. Awareness regarding *H. pylori* infections in Saudi Arabia awaits investigation to reduce or even eliminate the infection that would ease the substantial burden of managing *H. pylori* among both malignant and non-malignant diseases. *Aims*: The study aims were to (1) assess the knowledge of *H. pylori* infection, testing, and management among undergraduate students in Saudi Arabia and (2) compare the *H. pylori* knowledge among health science and non-health science students. *Methods:* This study involved a cross-sectional online survey among 334 undergraduate students in health science and non-health science colleges at King Saud University, Saudi Arabia, using a valid and reliable author-developed survey. The survey had two sections: the socio-demographic factors and knowledge items regarding *H. pylori*. Data were collected during the 2019–2020 academic year. Data analysis included descriptive statistics, Chi-square, and Mann–Whitney U test. The knowledge scores were categorized as poor, fair, and good. *Results:* Less than 10% of the students in both groups had a good knowledge level about *H. pylori.* The comparison of the overall mean between both groups was non-significant. Moreover, the level of knowledge of the respondents was significantly associated with their university level (*p* < 0.001), family monthly income (*p* < 0.007), having heard about *H. pylori* infection (*p* < 000.1), and a previous history of *H. pylori* infection (*p* < 000.1). *Conclusion:* The overall knowledge level of Saudi undergraduate students about *H. pylori* infection was low. Thus, health awareness interventions through educational programs are recommended for improving their knowledge about *H. pylori* infection and its prevention.

## 1. Introduction

*Helicobacter pylori* (*H. pylori*) are human pathogens transmitted from human to human through oral routes and cause chronic gastritis in all colonized subjects [1]. This gram-negative bacterium is a common infectious pathogen that inhabits the gastric mucosa in around 40–50% of the world’s population, leading to a global public health issue. The incidence of infection varies geographically and is over 75% in Portugal, Turkey and Kazakhstan, and some African countries [1,2]. In the Riyadh region of Saudi Arabia, Alghamdi et al. [3] reported an incidence of 34.7%. Furthermore, a recent review that included published literature from Saudi Arabia reported a prevalence ranging from 27–66% in the Middle East and North Africa region [4]. The high incidence and prevalence of *H. pylori* infection is a public health concern.

Evidence shows that *H. pylori* infection is associated with gastric cancer [5], the third leading cancer-related death and the fifth most common cancer worldwide [6]. It is also established that *H. pylori* is primarily related to the development of gastroduodenal disorders and, more commonly, chronic gastritis, peptic ulcers, and gastric adenocarcinoma, or lymphomas [7,8]. *H. pylori* gastritis is designated as an infectious disease in the Maastricht V/Florence consensus report [9]. The infection caused by *H. pylori* may either be asymptomatic and/or symptomatic. Epigastric pain, upper abdominal discomfort, indigestion, nausea, loss of appetite, reflux, and/or belching are among the most frequently occurring symptoms.

Commonly transmitted through oral routes, the fecal-oral, oral-oral, and gastro-oral routes, the definite interpersonal mode of transmission is still unknown [10,11]. However, several demographic factors are associated with the *H. pylori* infection. High income and higher educational levels are associated with a decrease in the prevalence of *H. pylori* gastritis [12,13]. Additionally, age, occupation, the type of drinking water used, consumption of fruits, vegetables, or fried food are also independent risk factors for *H. pylori* infection [14,15,16]. Smoking and alcohol consumption were also found to be independently positively associated factors [17]. Many of these risk factors are preventable with adequate knowledge and appropriate practice. There appears to be limited knowledge about *H. pylori* among the general population, primarily related to transmission. Evidence shows low awareness of *H. pylori* infection in Canada [18], the United States [19], China [20,21,22], and South Korea [23,24]. Knowledge about screening and prevention among risk groups, early diagnosis, and treatment may prompt people to seek measures to prevent the infection [9,24]. It is essential to educate the general population, especially the university students who are a segment of the general population.

There is a paucity of studies on *H. pylori* among university students who may be vulnerable to the infection. Therefore, assessing their awareness regarding *H. pylori* will form a basis for developing educational content that can enhance their knowledge and improve screening practices among those at risk. A comparison between health science and non-health science students will provide an understanding of the depth of knowledge they possess from their courses. To our knowledge, this is the first study that compares the knowledge about *H. pylori* infection, testing, and management among health science and non-health science undergraduate students in Saudi Arabia.

## 2. Methods

### 2.1. Study Design and Setting and Participants

This cross-sectional descriptive comparative study, a part of a large study, was designed to compare the knowledge of health science and non-health science undergraduate students regarding *H. pylori* in King Saud University, Riyadh, Saudi Arabia. Two health science colleges: College of Applied Medical Science and College of Nursing, and two non-health science colleges: College of Science and College of Computer Science were selected conveniently based on the accessibility of the researchers. The sample size calculated, using Raosoft, Inc., with 95% confidence and assuming an error of 5%, was 255. Compensating for nonresponse, 334 undergraduate students were selected. They were included if they had registered for the full-time baccalaureate program during the 2019–2020 academic year and consented to participate. Students enrolled in the common preparatory year were excluded.

### 2.2. Data Collection

Data were collected over six months, from September 2019 to February 2020, using a structured online survey on knowledge of *H. pylori*, developed by the authors. Six content experts validated the draft survey. Minor modifications were made as per their suggestions. The CVI index of the instrument was 0.915, ascertaining the validity of the questions. The final instrument had two sections: The first section assessed the socio-demographic factors, and the second section measured knowledge regarding *H. pylori* through 14 single-response multiple-choice items. The content areas of the survey were “meaning/perception of *H. pylori* infection and complications” (three items; items 1, 13, and 14), “signs and symptoms and risk factors” (four items; items 2–5), “mode of spread and prevention” (two items; items 6 and 8), “diagnosis, treatment, and self-care” (five items; items 7, 9–12). Each correct response was awarded a score of 1, with a total maximum possible score of 14. The knowledge scores were classified into three levels, based on the percentage of scores obtained by the participants: good (>75%), fair (50–75%), and poor (<50%). The questionnaire had excellent reliability (alpha = 0.949).

### 2.3. Procedure for Data Collection

After seeking ethical approval, the web link to the knowledge questionnaire, prepared using Google forms, was sent to the Questionnaire Centre of the University, which sent the invitation to the registered email of the students. The students were aware of the study’s purpose and that the data would be used only for research purposes. Participation was voluntary and responding to the questionnaire implied consent. Once the students clicked on the web link, they would view the information about the purpose of the study and the implied consent and would proceed by responding to the demographic items and the knowledge questions before clicking on the “submit” button. On average, participants completed the questionnaire in 15–20 min.

### 2.4. Ethical Consideration

The study received ethical approval from the Institutional Review Board, King Saud University (Ref. No. 21/0111/IRB). All data were kept confidential and were available only to the research team.

### 2.5. Statistical Analysis

The data were analyzed using the Statistical Package for Social Sciences (SPSS) version 20 (Armonk, NY, USA) for IBM. Frequency and percentage were computed to quantify the socio-demographic data and the responses of the participants on the individual knowledge items. The knowledge scores were categorized as poor, fair, and good, based on the percentage of scores obtained. The Mann–Whitney U test was used to find the difference in the knowledge scores in the two groups, and Chi-square was computed to find the relationship between the socio-demographic variables and the knowledge scores.

## 3. Results

### 3.1. Socio-Demographic Characteristics of the Respondents

The majority of the respondents were females (55.7%), in the age group 20 to <30 years (63.2%). Most of the respondents were enrolled in health science colleges (56.6%), with the highest proportion in the second year (fourth level) of study (44%). Most of them (56%) had 4–7 siblings. Almost two-thirds (64.1%) indicated having a monthly family income of more than 10,000 Saudi riyals (1 USD = 3.77 SR).

With regards to the respondents’ habits, 309 (92.5%) reported drinking coffee or tea. Out of the 309, the majority (53.7%) indicated consuming 2–5 cups of coffee or tea per day. More than half of the respondents (59.9%) reported having bottled water as their source of drinking water. When asked if they knew *H. pylori* is a microbe that causes peptic ulcers, the majority answered correctly (76.9%) and most responded that they acquired the information from reading articles (62.3%). Only 60 respondents (18.0%) reported having a previous history of *H. pylori* infection, and out of the 60, 50 (90.0%) reported that it was treated (Table 1).

### 3.2. Comparison of Knowledge about H. pylori between Health Science and Non-Health Science Students

**Figure 1:**
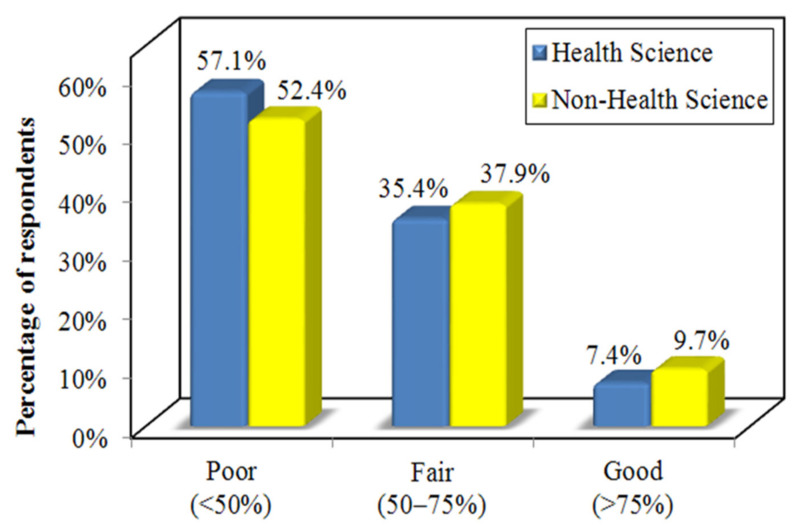
Bar graph showing the comparison of the level of knowledge scores among the health science and non-health science students.

The overall knowledge scores of health science and non-health science students about *H. pylori* infection were low, indicating poor knowledge levels in both groups. A slightly larger percentage (57.1%) of the health science students had poor knowledge compared to the non-health science students (52.4%). Furthermore, less than 10% of the students in both groups had good knowledge about *H. pylori* (Figure 1).

The highest mean was recorded among both groups in the item “*Helicobacter Pylori* (*H. pylori*) commonly found in the stomach is a bacterium” (0.71 in health science and 0.72 in non-health science groups). In contrast, the lowest mean score was recorded in both groups on the item related to the diagnosis of *H. pylori* infection. Only 24.9% in the health science group and 30.3% in the non-health science group responded correctly that a breath test was used to diagnose *H. pylori* infection, recording a mean of 0.25 and 0.30, respectively.

The comparison of the overall mean between the health science (44.44) and non-health science students (46.75) showed no significant difference (*p* = 0.361). Further, there was no significant difference between the groups on all items, except one: “*Which of the following is true regarding H. pylori infection*?” (Correct response: some people with the infection do not experience any signs and symptoms). The non-health science students had a higher mean score (0.42) on this item than the health science students (0.30). The difference was significant (*p* = 0.024), (Table 2).

### 3.3. Factors Accociated with the Knowledge about H. pylori among Respondents

Table 3 shows that the level of knowledge of the respondents was significantly associated with their university level (χ^2^ = 39.939, *p* < 0.001), family monthly income (χ^2^ = 17.658, *p* < 0.007), having heard about *H. pylori* infection (χ^2^ = 19.675, *p* < 000.1), and a previous history of *H. pylori* infection (χ^2^ = 20.116, *p* < 000.1). Knowledge on *H. pylori* was independent of all other socio-demographic characteristics of the students, including the field of study (health science and non-health science).

## 4. Discussion

This descriptive cross-sectional study compared the knowledge of *H. pylori* between health science and non-health science undergraduate students in Saudi Arabia and found that the overall level of knowledge was poor among both groups. Our finding of poor knowledge was similar to those reported among physicians and students in a national survey in China [22] and among the healthy population in Korea [24]. A lower level of knowledge (24.6%) was also reported among the general population in the United Arab Emirates (UAE) [25]. In our study, the largest proportion of students were in the younger age group (20–30 years) studying in health science colleges (56.6%), in the fourth level of their program (44%), and female students (55.7%). The variations in the respondents’ characteristics in this study and those in the Chinese, Korean, and UAE studies may explain the possible reasons for the differences in the knowledge level.

The current study also found a slightly higher level of knowledge (M = 46.75) in the non-health science students on *H. pylori* infection, compared to the health science students (M = 44.44); the difference was not significant (0.361). The apparently higher knowledge in the non-health science students could be attributed to the fact that a section of them had undergone courses in Zoology, Microbiology, Physiology, and Bacteriology. It is possible that these students may have been exposed to some information on *H. pylori* infection, thereby influencing their knowledge scores. This may also explain why health science students recorded a significantly lower mean score in one item (*p* = 0.024) *“Which of the following is true regarding H. pylori infection?”* to which students responded correctly that some people with the infection do not experience any signs and symptoms. Furthermore, a low emphasis on *H. pylori* infection in the curriculum of health science students may explain the difference. This study found that, overall, students recorded the highest mean (0.71) for the item “*H. pylori* is commonly found in the stomach is a bacterium” and the least mean (0.25) on the item “*H. pylori* can be diagnosed by examination of breath test”. The findings suggest that students know that *H. pylori* is a disease related to the gastrointestinal system. Nevertheless, they had limited awareness of how the condition is diagnosed. Similarly, a lack of knowledge regarding diagnostic and treatment recommendations for *H. pylori* was also recorded in a study by Cano-Contreras et al. [26] among primary care physicians in Mexico. It is therefore not surprising that our respondents had a low level of knowledge regarding diagnosis. Future educational interventions should focus on this aspect of *H. pylori* awareness.

A lower level of knowledge ranging from 23.8–31.1% was recorded in Chinese and Korean communities regarding the route of *H. pylori* transmission [20,21,24], compared to the undergraduate health science (42.3%) and non-health science (49%) students in the current study. The low level of education (high school level to high school graduate) in the Chinese and Korean population, compared to the respondents in our study who are university students, may explain the reason for the differences.

With regards to symptoms and complications of *H. pylori* infection, Shin et al. [24] reported correct responses on knowledge items on symptoms in 37.2% and complications in 58.3% of healthy Korean adults. These figures are lower than our findings that recorded correct response in two items of symptoms 47.1% and 54.5% of health science and in 39.3% and 58.6% of non-health science students. Regarding complications, correct responses were found in 38.6% health science and 36.6% non-health science students.

Interestingly, the university level was significantly associated with the respondents’ knowledge level about *H. pylori* infection. A previous study among the general population in Cameroon also showed a significant association between level of education and knowledge on *H. pylori* infection among participants. However, most of them had tertiary education and only 20.9% among them had a low level of knowledge on *H. pylori* [27]. Similarly, in the present study, a low proportion of students (31.2%) who had progressed to the fourth year (in level 8) had poor knowledge compared to the large proportion (70.1%) in the second year (level 2) of study, suggesting that they have acquired knowledge on *H. pylori* with advancement in educational levels. Moreover, the level of education was associated with knowledge on *H. pylori* infection among the general population who were screened in Cameroon [27].

Concerning family monthly income, a significant association was found with the level of knowledge on *H. pylori* infection. Most of the respondents (64.1%) in this study were from Class 3 or a high socioeconomic status (family income > 10,000 SR; 1 USD = 3.77 SR). A family in Saudi Arabia earning between 10,000 to 15,000 SR belongs to Class 3 socioeconomic status; the highest being Class 4 (above 15,000 SR) [28]. In parallel, a study in China showed that people with medium to low levels of family income have poor knowledge regarding *H. pylori*, besides having a high infection rate [21].

This study also found a significant association between the item that assessed whether the students had heard about *H. pylori,* and their knowledge level. Undeniably, *H. pylori* infection is highly prevalent in developing countries whereas in Saudi Arabia it is hyperendemic [29]. The Ministry of Health in Saudi Arabia has an electronic media interface that publishes information on health issues through the awareness platform, which is accessible to the public, including students. Furthermore, a large proportion of our respondents reported “reading articles” and “media” as their main sources of information. This may explain why most of the students (76.9%) reported having heard about *H. pylori*, which is a higher proportion than those reported among Chinese physicians and the general population (49.8%) [22] and those reported in a review of nine studies from China, South Korea, North America, Ethiopia, India, and Malaysia (22–33%). Eight of the nine studies were conducted among the population at risk, except North America, where they were from the general population. Variations in the population characteristics may also account for the differences between our findings and those of previous studies [30].

The respondents’ previous history of *H. pylori* infection was significantly associated with their knowledge levels. Often, people have limited knowledge of the nature of the disease and its complications [31]. However, the experience of an illness may have an influence on their knowledge through interactions with health personnel, especially when they undergo treatment. In total, 90% (54 out of 60) had undergone treatment for *H. pylori* infection and may have acquired knowledge during their treatment. This study did not find an association between all other socio-demographic factors (age, gender, number of siblings), including the field of university study (health science versus non-health science) and knowledge of *H. pylori*, suggesting that these factors are independent of *H. pylori* knowledge among university students. Future studies should explore these factors in larger samples.

## 5. Strengths and Limitations

The strength of this study is that it is the first to compare the knowledge of health science and non-health science university students about *H. pylori* in Saudi Arabia. However, this study is not without limitations. Participation in the online survey was voluntary. Hence most of the participants may have been interested in the subject, leading to sampling bias. The study was conducted in one university, therefore limiting the generalizability of the findings. Previous studies [20,21,32,33] found that handwashing practices before eating a meal and after using the toilet, safe food practices, and drinking water from a clean source were associated with less *H. pylori* infection. Knowledge on these aspects was not assessed in the current study. Further, assessing classroom or school education as a source of *H. pylori* information would provide evidence on whether students had acquired knowledge about *H. pylori* infection before embarking on their university education. Future studies should consider including these aspects to explore sources of information. We assessed the students’ knowledge using a 14-item questionnaire developed by the authors. Using this survey, a small percentage of students who had responded “No” to the item “Have you hear about *H. pylori* infection (a microbe which causes peptic ulcers?)” provided responses to other knowledge items in the questionnaire. Although the questionnaire was validated and the reliability established, we attribute the response of the item to the chance factor in survey studies.

## 6. Conclusions

Saudi undergraduate students have relatively low knowledge about *H. pylori*, particularly among health science and non-health science students. Low *H. pylori* knowledge has been reported globally. Future studies on larger samples and varied populations will be of significant value to identify knowledge gaps. Raising health awareness regarding *H. pylori* infection is essential for effectively implementing population-based *H. pylori* screening and treatment programs. Moreover, interventions, including health awareness educational programs, would increase adherence to advice to seek medical attention. The findings of this study will form the basis for developing educational programs.

## Figures and Tables

**Table 1:** Socio-demographic characteristics of the respondents (*n* = 334).

Items	Total Sample (*n* = 334)		Health Science (*n* = 189)		Non-Health Science (*n* = 145)	
	*n*	%	*n*	%	*n*	%
Age (Years)						
<20	94	28.1	52	27.5	42	29.0
20–30	211	63.2	116	61.4	95	65.5
≥30	29	8.7	21	11.1	8	5.5
Gender						
Male	148	44.3	68	36.0	80	55.2
Female	186	55.7	121	64.0	65	44.8
Field of study in the university						
Health colleges	189	56.6	189	56.6	-	-
Applied Medical Sciences	132	39.5	132	39.5	-	-
Nursing	57	17.1	57	17.1	-	-
Non-Health colleges	145	43.4	-	-	145	43.4
Sciences	80	24.0	-	-	80	24.0
Computer Sciences	65	19.5	-	-	65	19.5
University level						
Level 4 (Year II, Semester 2)	147	44.0	83	43.9	64	44.1
Level 5 (Year III, Semester 1)	38	11.4	24	12.7	14	9.7
Level 6 (Year III, Semester 2)	50	15.0	23	12.2	27	18.6
Level 7 (Year IV, Semester 1)	22	6.6	16	8.5	6	4.1
Level 8 (Year IV, Semester 2)	77	23.1	43	22.8	34	23.4
No. of siblings at home						
No. siblings	11	3.3	6	3.2	5	3.4
<3	99	29.6	55	29.1	44	30.3
4–7	187	56	106	56.1	81	55.9
>7	37	11.1	22	11.6	15	10.3
Family monthly income (1 USD = approx. 3.77 SR)						
Less than 2500 SR	16	4.8	10	5.3	6	4.1
2500–5000 SR	31	9.3	18	9.5	13	9.0
5000–10,000 SR	73	21.9	38	20.1	35	24.1
More than 10,000 SR	214	64.1	123	65.1	91	62.8
Do you drink coffee or tea?						
Yes	309	92.5	171	90.5	138	95.2
No	25	7.5	18	9.5	7	4.8
If yes, how many cups per day? (*n* = 309)						
Less than 2 Cups	126	40.8	67	39.2	59	42.8
2–5 Cups	166	53.7	93	54.4	73	52.9
More than 5 Cups	17	5.5	11	6.4	6	4.3
What sources of drinking water does your family use?						
Tap water	79	23.7	41	21.7	38	26.2
Bottled water	200	59.9	114	60.3	86	59.3
Both	55	16.5	34	18.0	21	14.5
Do you hear about *H. pylori* infection (a microbe which causes peptic ulcers)?						
Yes	257	76.9	155	82.0	102	70.3
No	77	23.1	34	18.0	43	29.7
If yes, what is the source of your knowledge? (*n* = 257) *						
Not applicable	20	7.8	12	7.7	8	7.8
Family	99	38.5	55	35.5	44	43.1
Mass media	106	41.2	62	40.0	44	43.1
Reading articles	160	62.3	100	64.5	60	58.8
Neighborhood	13	5.1	9	5.8	4	3.9
Did you have previous history of *H. pylori* infection?						
Yes	60	18.0	32	16.9	28	19.3
No	274	82.0	157	83.1	117	80.7
If yes, have you been treated? (*n* = 60)						
Yes	54	90.0	27	84.4	27	96.4
No	6	10.0	5	15.6	1	3.6

* More than one response.

**Table 2:** Knowledge of the respondents about *H. pylori* infection items (*n* = 334).

Questions/Items	Health Science (*n* = 189)				Non-Health Science (*n* = 145)				*p*
	Incorrect		Correct		Incorrect		Correct		
	*n*	%	*n*	%	*n*	%	*n*	%	
1. *Helicobacter Pylori* (*H. pylori*) commonly found in the stomach is a (Bacterium)	54	28.6	135	71.4	41	28.3	104	71.7	0.953
2. Which of the following is a sign/symptom of *H. pylori* infection? (Burning pain in the upper abdomen)	86	45.5	103	54.5	60	41.4	85	58.6	0.452
3. A person suffering from *H. pylori* infection will experience which of the following? (Nausea)	100	52.9	89	47.1	88	60.7	57	39.3	0.156
4. Which of the following is a risk factor for *H. pylori* infection? (Low socio-economic status)	130	68.8	59	31.2	93	64.1	52	35.9	0.372
5. Which of the following is NOT a risk factor for *H. pylori* infection? (Eating plenty of chilly and pepper)	108	57.1	81	42.9	78	53.8	67	46.2	0.542
6. *H. pylori* infection can be passed from one person to another through which of the following ways/routes? (Oral route)	109	57.7	80	42.3	74	51.0	71	49.0	0.228
7. *H. pylori* can be diagnosed by examination of (Breath test)	142	75.1	47	24.9	101	69.7	44	30.3	0.266
8. Which of the following is a measure to prevent *H*. *pylori* infection? (Washing hands)	91	48.1	98	51.9	75	51.7	70	48.3	0.518
9. If a person has *H. pylori* infection, he/she can be treated with (tablets)	98	51.9	91	48.1	76	52.4	69	47.6	0.919
10. The duration of treatment for *H. pylori* infection is (one to two weeks)	126	66.7	63	33.3	95	65.5	50	34.5	0.826
11. In order to avoid the recurrence of *H. pylori* infection, a person should (comply with the treatment plan)	84	44.4	105	55.6	63	43.4	82	56.6	0.856
12. If your friend or relative is suspected of having *H. pylori* infection, he/she should seek immediate medical advice in which of the following conditions? (Persistent burning pain in the upper abdomen)	94	49.7	95	50.3	61	42.1	84	57.9	0.164
13. Which of the following is true regarding *H. pylori* infection? (Some people with the infection do not experience any signs and symptoms)	132	69.8	57	30.2	84	57.9	61	42.1	0.024 *
14. What complication does *H. pylori* infection cause? (Gastric cancer)	116	61.4	73	38.6	92	63.4	53	36.6	0.699
Overall Mean	44.44				46.75				0.361

Note: Correct response is placed in parenthesis. * Statistically significant at *p* ≤ 0.05.

**Table 3:** Relationship between the socio-demographic characteristics and the knowledge of the respondents about *H. pylori* infection (*n* = 334).

Q	Socio Demographic Data	Knowledge about *H. pylori* Infection						*p*
		Poor <50% (*n* = 184)		Fair 50–75% (*n* = 122)		Good ≥75% (*n* = 28)		
		No.	%	No.	%	No.	%	
**1**	Age (Years)							
	<20	52	55.3	32	34.0	10	10.6	0.905
	20–<30	116	55.0	79	37.4	16	7.6	
	≥30	16	55.2	11	37.9	2	6.9	
**2**	Gender							
	Male	84	56.8	57	38.5	7	4.7	0.098
	Female	100	53.8	65	34.9	21	11.3	
**3**	Field of study in the university							
	Applied Medical Sciences	76	57.6	50	37.9	6	4.5	0.227
	Nursing	32	56.1	17	29.8	8	14.0	
	Sciences	40	50.0	34	42.5	6	7.5	
	Computer Sciences	36	55.4	21	32.3	8	12.3	
**4**	University level							
	Level 4 (Year II, Semester 2)	103	70.1	39	26.5	5	3.4	<0.001 ^*^
	Level 5 (Year III, Semester 1)	20	52.6	17	44.7	1	2.6	
	Level 6 (Year III, Semester 2)	28	56.0	17	34.0	5	10.0	
	Level 7 (Year IV, Semester 1)	9	40.9	9	40.9	4	18.2	
	Level 8 (Year IV, Semester 2)	24	31.2	40	51.9	13	16.9	
**5**	No. of siblings at home							
	No. siblings	7	3.8	3	2.5	1	3.6	0.964
	<3	52	28.3	39	32.0	8	28.6	
	4–7	106	57.6	65	53.3	16	57.1	
	>7	19	10.3	15	12.3	3	10.7	
**6**	Family monthly income (1 USD = approx. 3.77 SR)							
	Less than 2500 SR	11	68.8	2	12.5	3	18.8	0.007 ^*^
	2500–5000 SR	24	77.4	6	19.4	1	3.2	
	5000–10,000 SR	46	63.0	22	30.1	5	6.8	
	More than 10,000 SR	103	48.1	92	43.0	19	8.9	
**10**	Have you hear about *H. pylori* infection (a microbe that causes peptic ulcers?)							
	Yes	126	49.0	103	40.1	28	10.9	<0.001 ^*^
	No	58	75.3	19	24.7	0	0.0	
**12**	Do you have previous history of *H. pylori* infection?							
	Yes	20	33.3	28	46.7	12	20.0	<0.001 ^*^
	No	164	59.9	94	34.3	16	5.8	

Note: Q = Questions. * Statistically significant at *p* ≤ 0.05.
